# Generation of A Space-Variant Vector Beam with Catenary-Shaped Polarization States

**DOI:** 10.3390/ma15082940

**Published:** 2022-04-18

**Authors:** Junjie Wang, Mingbo Pu, Jinjin Jin, Fei Zhang, Ling Liu, Weijie Kong, Xiong Li, Yinghui Guo, Xiangang Luo

**Affiliations:** 1 State Key Laboratory of Optical Technologies on Nano-Fabrication and Micro-Engineering, Institute of Optics and Electronics, Chinese Academy of Sciences, Chengdu 610209, China; wangjunjie_w6@163.com (J.W.); pmb@ioe.ac.cn (M.P.); ioejinjinjin@163.com (J.J.); zhangfei_ns@163.com (F.Z.); lling09@126.com (L.L.); kongwj@ioe.ac.cn (W.K.); qiling006@163.com (X.L.); guoyinghui8@163.com (Y.G.); 2 School of Optoelectronics, University of Chinese Academy of Sciences, Beijing 100049, China

**Keywords:** catenary, polarization interferometry, vector beams, liquid crystals

## Abstract

We demonstrate the generation of a space-variant vector beam with catenary-shaped polarization states based on the polarization interferometry. With a spatial light modulator and a common path interferometric configuration, two orthogonally circularly polarized beams with different phase modulation overlap each other, yielding the vector beams. In addition, the polarization states of this vector beam are scalable to the arbitrary spatial distribution because of its great flexibility and universal applicability. It is expected that this vector beam may have many potential and intriguing applications in the micro/nano material processing, liquid crystal elements fabrication and optical micro-manipulation, and so on.

## 1. Introduction

According to the spatial distribution of states of polarization (SOPs), the beams can be classified into homogeneously polarized beams and non-homogeneously polarized beams, which are also known as scalar beams and vector beams. The majority of previous researchers focus on scalar beams, such as linearly, elliptically, circularly, and partially polarized. In recent years, there has been an increasing interest in manipulating polarization, owing to the fascinating properties of vector beams. For instance, cylindrical vector beams are a relatively special but common class of non-homogeneously polarized beams [1], known for cylindrical symmetry in polarization and are of great interest to scholars because of their unique focusing characteristics with a high numerical aperture [2,3]. In particular, there is a strong longitudinal electric field component at the focus of the radially polarized (RP) beam [4], leading to a sharper focal spot than scalar beams [5]. As a contrast, a bright focal ring with zero intensity at the center can be obtained when an azimuthally polarized (AP) beam is focused. Owing to their unique properties, vector beams have many potential and intriguing applications in high-resolution microscopy imaging [6], particle acceleration [7], material processing [8], laser microprocessing [9], and so on. It is also noteworthy that manipulating polarization of vector beams is beneficial to the fabrication of liquid crystal elements [10,11]. Nematic liquid crystals have a natural advantage in fabricating geometric phase elements because of rotatable rod-like liquid crystal molecules, self-organizing properties, and dielectric anisotropy of liquid crystal materials [12]. A linear photoalignment polymer, inducing nematic liquid crystal molecules to align as a phototropic material sensitive to polarization, can record a linear polarization orientation map of vector beams, which enables that long axis profile of nematic liquid crystal molecules to follow the orientation map. Under these circumstances, manipulating polarization distribution of vector beams means controlling the arrangement of nematic liquid crystal molecules, which is a precondition to utilize vector beams for fabricating liquid crystal elements. There are two main fabrication methods for liquid crystal elements: direct-write and interferometer [10]. The direct-write method is equivalent to the realization of light source with arbitrary predetermined polarization distribution, employing a direct-write laser scanner to reset the SOP at each point in the cross section. Although the direct-write method has great flexibility, it simultaneously introduces more complicated mechanical experimental equipment. The interferometer method employs a modified Mach–Zehnder interferometer to generate the vector beams for fabricating liquid crystal elements. Liquid crystal elements can record the wavefront of the physical optical elements placed at the optical path of the primary beam, such as geometric phase gratings and geometric phase lenses. Due to limited physical optical elements and unstable experiment setup caused by physical optical element replacement, this method severely limits the flexibility of application and the diversity of liquid crystal elements. Therefore, it is necessary to study the methods of generating arbitrary vector beams, which are suitable for more liquid crystal elements fabrication under one-shot exposure.

Numerous methods for generating vector beams have been studied and developed, particularly in the past 20 years [13,14]. Depending on whether the generation methods involve gain media, these methods can be categorized as active or passive. To generate the desired vector beams, active methods involve adding certain characteristic optical components to the resonant cavity of the laser, such as birefringent crystals. Because of the high cost and the requirement to create complex optical systems, the active methods are less used. To tackle this difficulty, researchers proposed passive methods, which involve relocating the complicated optical system inside the laser resonant cavity to free space to generate vector beams. Because they are independent of the gain media and enable real-time operation of the extra-cavity experimental setup, passive methods are widely employed in the generation of vector beams. Axial birefringent elements [15], helical phase plates [16], conical Brewster prisms [17], spatially variable retardation plates [18], space-variant dielectric subwavelength gratings [19], diffractive liquid crystals device [20,21], and metasurfaces are some of the most often utilized passive methods. In particular, metasurfaces [22,23,24,25,26,27,28,29,30,31,32,33,34], which are a novel type of subwavelength structures that enable precise modulation of phase, amplitude, and polarization of electromagnetic waves [35,36,37,38], have attracted a lot of attention from researchers in recent years. Recently, the combination of metasurface and liquid crystals has also developed novel and interesting applications [39]. These passive methods generally generate vector beams of specific types or polarization distributions according to predetermined phase profiles. In other words, the above methods lack flexibility and have high demands on sophisticated micro/nano fabrication technologies. To meet the requirements of the generation of arbitrary vector beams, researchers introduce a spatial light modulator (SLM), which allows for the flexible construction of arbitrary spatial modulation (phase, or/and amplitude) patterns to generate the appropriate optical modes, opening up the possibility of generating arbitrary vector beams [40,41,42]. In this article, we make use of the advantage of the SLM’s flexibility and polarization sensitivity of liquid crystals to generate a space-variant vector beam with catenary-shaped polarization states. This vector beam is beneficial to the fabrication of liquid crystal elements with the desired liquid crystal molecule arrangement through manipulating the polarization distribution of the vector beam. As such, the catenary-shaped polarized beam has promising prospects to fabricate the optical nanostructures and liquid crystal elements. Furthermore, the SOPs of this vector beam are scalable to the arbitrary spatial distribution because of its great flexibility, which greatly activates many creative ideas for researching novel effects and phenomena of full vector beams.

## 2. Experimental Setup

**Figure 1:**
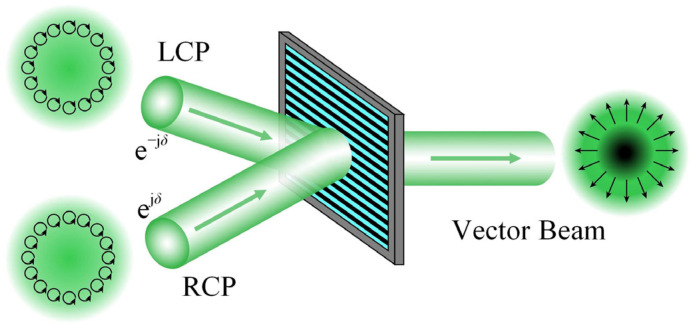
A space-variant linearly polarized beam is generated by the superposition of the left and right circularly polarized beams with inverse phase profiles. The left and right circularly polarized beams overlap each other in the grating, and coaxially combine into the vector beam.

**Figure 2:**
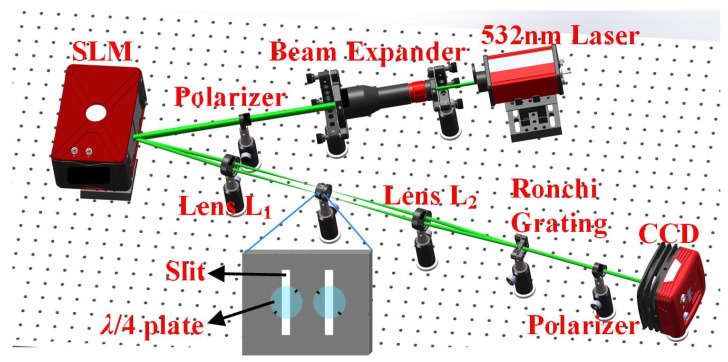
Schematic of experimental setup composed of an SLM and a 4-*f* system for generating vector beams.

The spatial SOPs of a vector beam, unlike homogeneously polarized beams, may be flexibly adjusted to the desired distribution. According to the polarization interferometry, the phase retardation between two orthogonally circularly polarized (left circular polarization, LCP, and right circular polarization, RCP) scalar beams with inverse phase profiles can be utilized to regulate SOPs distribution of the interference pattern, generating a space-variant linearly polarized beam, as shown in Figure 1. More generally, if the phase retardation at each location of the two orthogonally circularly polarized beams is modified freely, an interference pattern with any predetermined SOPs distribution will be configurated for generating arbitrary vector beams. It is also a practicable approach to arrange a common path interferometric configuration assisted by a 4-*f* system and an SLM. The experimental setup is shown in Figure 2.

Firstly, a 532 nm collimated laser beam, passing through a linear polarizer in the *x* direction, illuminates a reflective liquid crystal SLM which is equipped with 1920 × 1080 pixels (each pixel has an 8 μm × 8 μm size). Here the diameter of the collimated laser beam has been enlarged by the beam expander to match the panel window size of the SLM. In the 4-*f* system, composed of a pair of identical lenses (L_1_ and L_2_) with the same focal length of *f*, SLM and Ronchi phase grating are placed at the front focal plane of L_1_ and the rear focal plane of L_2_, respectively. The rear focal plane of L_1_ and the front focal plane of L_2_ overlap at the middle plane of the 4-*f* system, which is known as the Fourier plane of the 4-*f* system. Secondly, the incident linearly polarized beam is diffracted into various diffraction orders by a predetermined holographic grating (HG) displayed at the SLM. A spatial filter with two separate slits is placed at the Fourier plane to only let the beams with ±1-orders pass through. Two *λ*/4 plates with long axes perpendicular to each other are located immediately behind the two slits, converting the beams with ±1-orders into left and right circularly polarized beams, respectively. The inset in Figure 2 shows the real setup of the two slits of the spatial filter and the two *λ*/4 plates. The short black lines denote the long axes orientations of the two *λ*/4 plates. Then, the left and right circularly polarized beams are recombined into a space-variant linearly polarized beam at the Ronchi phase grating, which is inserted at the rear focal plane of L_2_, based on the polarization interferometry. The two circularly polarized beams can interfere at the Ronchi phase grating that enable a coaxial and collimated vector beam. The period of the HG is adjusted to the same as that of the Ronchi phase grating in this case. To calibrate the optical path difference between the two combined beams, a detailed explanation is necessary. When the linearly polarized beam in the *x* direction illuminates the SLM, the beam with 0-order of the reflected beams is not endowed with additional phase factor. We utilize the beam with 0-order as a standard reference beam to examine the collimation of optical path of the 4-*f* system, because the collimation of optical path enables no additional optical path difference introduced. Here we adjust frequently the optical path until the output beam with 0-order becomes collimated, with the help of a shear interferometer. After that, we continue to fine-tune the optical path to ensure that the diameters of the two beams with ±1-orders are the same, which indicates that the optical path difference of the two beams has been compensated. Finally, a color Charge Coupled Device (CCD) is positioned behind the Ronchi phase grating to receive generated vector beams.

Dissecting the entire optical configuration along the propagation direction of the beams, a linearly polarized beam incident on the SLM can be generated by extending, collimating, and polarizing a 532 nm laser beam. The complex amplitude reflectance function of the HG displayed at the SLM can be written as follows:

(1) $$r(x,\;y)=\frac{1}{2}\left[1+\gamma \cos(2\pi f_{0}x+\delta )\right]=\frac{1}{2}+\frac{\gamma }{4}e^{j(2\pi f_{0}x+\delta )}+\frac{\gamma }{4}e^{-j(2\pi f_{0}x+\delta )}$$

where *δ* is the additional phase factor, and *f*_0_ and *γ* are the spatial frequency and modulation degree of the HG, respectively. It is obvious that the beams with the ±1-orders feature diffraction energy with a reflection coefficient of *γ*/4 and a tunable phase factor $e^{\pm j(2\pi f_{0}x+\delta )}$ proportional to e^±j*δ*^. Therefore, it is the beams with ±1-orders that are available for generating vector beams in the subsequent optical configuration. Assume that *p* is the period of the HG as follows:

(2) $$p=\frac{1}{f_{0}}=\frac{\lambda }{\sin(\theta )}$$

where *θ* is the diffraction angle of the beams with ±1-orders, and *λ* is the wavelength. Assume that E*_in_*(*x*, *y*) = E_0_(*x*, *y*)[10]^T^ is the linearly polarized beam in the *x* direction incident on the SLM, then the reflected beams after the SLM can be written as follows:

(3) $$E_{out}(x,y)=\frac{1}{2}E_{0}(x,y)\left(\begin{array}{l} 1\\ 0 \end{array}\right)+\frac{\gamma }{4}e^{j2\pi f_{0}x}e^{j\delta }E_{0}(x,y)\left(\begin{array}{l} 1\\ 0 \end{array}\right)+\frac{\gamma }{4}e^{-j2\pi f_{0}x}e^{-j\delta }E_{0}(x,y)\left(\begin{array}{l} 1\\ 0 \end{array}\right)$$

As we can see, all of the reflected beams are still linearly polarized beams in the *x* direction; in particular, the beams with ±1-orders are endowed with additional phase factors e^±j*δ*^ that can be freely modulated by the SLM. According to the polarization interferometry, we simplify the amplitude description because the beams with ±1-orders have the same square of the two amplitudes. After passing through two slits and two *λ*/4 plates, the beams with ±1-orders are converted into the right and left circularly polarized beams, respectively as follows:

(4) $$E^{+1}(x,y)=\frac{e^{j\delta }}{2}\left(\begin{array}{l} 1\\ -j \end{array}\right),\;E^{-1}(x,y)=\frac{e^{-j\delta }}{2}\left(\begin{array}{l} 1\\ j \end{array}\right)$$

Then, the right and left circularly polarized beams are collinearly recombined behind the Ronchi grating, generating a space-variant linearly polarized vector beam, which can be written as follows:

(5) $$E_{z}=\cos(\alpha )(E^{+1}+E^{-1})=\frac{1}{2}\cos(\alpha )\left(\begin{array}{l} e^{j\delta }+e^{-j\delta }\\ {-\mathrm{je}}^{j\delta }+{\mathrm{je}}^{-j\delta } \end{array}\right)=\cos(\alpha )\left(\begin{array}{l} \cos(\delta )\\ \sin(\delta ) \end{array}\right)$$

where *α* is the angle between the propagation direction of the beams with ±1-orders and the normal line of the Ronchi grating. Here cos(*α*) is approximately equal to 1 since *α* is quite small and satisfies the paraxial condition. Evidently, the above expressions show that arbitrary polarization beams can be generated by choosing an appropriate phase *δ* regulated by the SLM. Most previous researchers are interested in a helical phase profile of *δ* = *lφ* + *φ*_0_, where *l* is the topological charge, *φ* is the azimuthal angle, and *φ*_0_ is the initial phase. Of course, many unattempted phase profiles, except for the helical phase profile, should be focused on and studied. Therefore, we also employ and experiment with the original quadratic phase and the quadratic phase superimposed with different topological charges, yielding a space-variant vector beam with catenary-shaped polarization states. Encouragingly, generating this catenary-shaped vector beam may be a huge boost for fabricating the optical nanostructures and liquid crystal elements.

## 3. Analysis of the Design Principle

**Figure 3:**
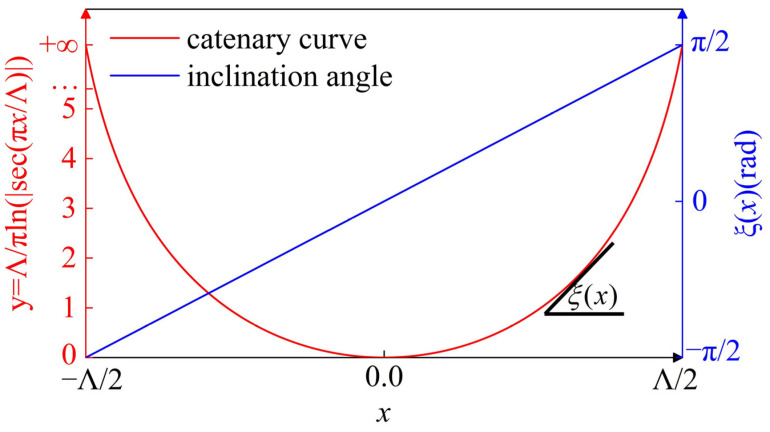
Catenary curve (red) and inclination angle (blue) distributions in a single catenary.

In order to explore more vector beams with unattempted phase profiles, we consider the original quadratic phase of catenary elements as a reference phase profiles. For geometric phase catenary elements, a catenary-shaped nanostructure arrangement has a continuous and excellent phase modulation effect. Here, we illustrate the relevant principles of the catenary and the phase modulation. The catenary is a curve formed by a flexible wire, rope, or chain hanging freely from two separate points. Under equilibrium, the tension and gravity force would cancel in both the horizontal and vertical directions. The catenary of equal strength was first derived in 1826 by Davies Gilbert as follows [43]:

(6) $$y=\frac{\Lambda }{\pi }\ln(|\sec(\pi x/\Lambda )|)$$

where Λ is the horizontal length of a single catenary. Recently, the catenary was applied to optics [44], and a new area called Catenary Optics has formed [45]. As we know, although phase is controlled by an optical path difference in optics and photonics, there is a common and alternative phase control scheme in the subwavelength optics: the geometric phase shift. For example, geometric phase subwavelength elements can achieve geometric phase shift by the rotation angle of a nanostructure. When the nanostructure is catenary-shaped, it can be considered as a continuous catenary structure consisting of many small nanostructures. We show a schematic of the catenary curve and the inclination angle of its tangent in a two-dimensional coordinate system, as shown in Figure 3.

The inclination angle between the curve tangent and the x axis, *ξ*(*x*) varies consecutively from −*π*/2 to *π*/2 between the left and right endpoints. According to the geometric phase relation, the catenary structure with continuous inclination angle variability can yield a linear geometric phase as follows:

(7) Φ(x)=2σξ(x)

where *σ* = ±1 denotes the left and right circular polarizations. The geometric phase, also known as Pancharatnam–Berry phase (PB phase) [46], derives from the coupling between the intrinsic angular momentum of the photon and the rotation of the coordinate system, and is thus considered as a result of the photonic spin–orbit interactions [47]. Notably, different from the discrete sampled phases of some subwavelength structures, the geometric phase in the catenary structure is linearly continuous when the small nanostructure has half-wave retardation as a local half-wave plate. For liquid crystal materials, the half-wave condition can be written as follows:

(8) $$\Delta nd=(m+\frac{1}{2})\lambda$$

where Δ*n* is birefringent indexes of liquid crystal materials, *d* is the effective thickness of the liquid crystal layer, and *m* is the natural number. When the thickness of the liquid crystals satisfies the half-wave condition at a certain operating wavelength, liquid crystal molecules can also be considered as local half-wave plates. The effective thickness of the liquid crystal layer can be adjusted by an external electric field to satisfy the half-wave condition of different wavelengths, which is much more flexible than the subwavelength elements operating at a single wavelength. Furthermore, the dosage of one-shot exposure is about 1 J/cm^2^ and is capable of reducing the difficulty of the experiment [11]. Liquid crystals, as a phototropic material sensitive to polarization, can record the spatial distribution of polarization states of the vector beam, which is appropriate for fabricating liquid crystal elements with one-shot exposure. Therefore, the liquid crystal elements, imitating geometric phase subwavelength catenary elements, have not only an excellent phase modulation effect, but also a great flexibility and a low fabrication complexity. On the basis of this, we adopted the idea of designing the full structure of geometric phase subwavelength catenary elements to generate the vector beams with catenary-shaped polarization states. Spatially manipulating the SOPs of the vector beam is expected to be the same as the catenary by matching nanostructures arrangement of catenary elements. According to the geometric phase, nanostructure can yield the geometric phase strictly proportional to its inclination angle. Therefore, the desired phase profile δ displayed at the SLM can be calculated and derived from the predetermined phase profile of catenary elements. Without loss of generality, the representative catenary elements are selected: a wide-angle metalens and a wide-angle metalens carrying orbital angular momentum (OAM). The quadratic phase, as the predetermined phase profile of the wide-angle metalens, can be generally written as follows:

(9) $$\Phi (r)=-\frac{k_{0}r^{2}}{2f_{0}}+\pi$$

where *k*_0_ is the wavenumber in free space, *f*_0_ is the corresponding focal length, and the initial phase is set as *π* [48,49]. According to Equation (7), the desired phase profile displayed at the SLM, mapped by the predetermined phase profile of the wide-angle catenary metalens, can be written as follows:

(10) $$\delta_{1}=\xi (r)=\frac{\Phi (r)}{2}=-\frac{k_{0}r^{2}}{4f_{0}}+\frac{\pi }{2}$$

We also investigated the quadratic phase carrying OAM. That is, the helical phase with different topological charges is superimposed on the basis of the general quadratic phase, which can be written as follows:

(11) $$\Phi (r,\varphi )=-\frac{k_{0}r^{2}}{2f_{0}}+\pi +l\varphi$$

Similarly, the desired phase profile displayed at the SLM can be written as follows:

(12) $$\delta_{2}=\frac{\Phi (r,\varphi )}{2}=-\frac{k_{0}r^{2}}{4f_{0}}+\frac{l}{2}\varphi +\frac{\pi }{2}$$

The phase modulation of the incident beam can be realized by loading the desired phase profile as the form of hologram into the SLM.

## 4. Results and Discussion

**Figure 4:**
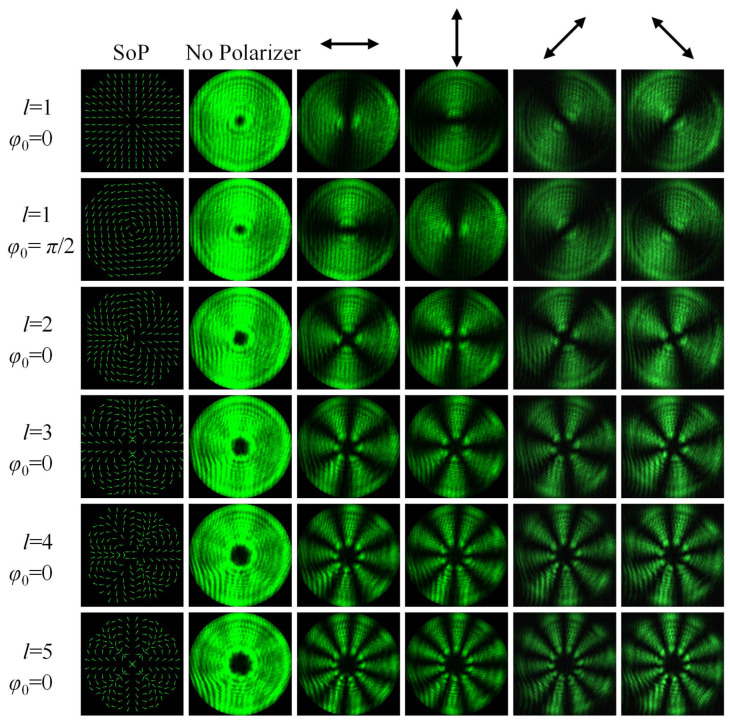
Generated vector beams with topological charges of *l* = 1, 2, 3, 4 and 5, respectively. The first two rows correspond to RP beam and AP beam for *φ*_0_ = 0 and *π*/2. The first column represents the theoretical SOPs distribution of different vector beams. The second, third, fourth, fifth and sixth columns represent the intensity patterns of the vector beams after inserting no polarizer, horizontal polarizer, vertical polarizer, 45° polarizer and 135° polarizer, respectively.

Firstly, we examine the generation of the vector beams in the helical phase with different topological charges *l*, as shown in Figure 4. In particular, there are two very representative vector beams for *l* = 1, RP beam and AP beam, corresponding to the initial phases *φ*_0_ of 0 and *π*/2, respectively. Evidently, we cannot distinguish the difference between the vector beam and the scalar beam from the intensity patterns when no polarizer is inserted. Different from intensity fringes of the conventional interference pattern of two identically polarized beams, the polarization interference pattern of two orthogonally polarized beams has no intensity fringes. However, we can identify two vector beams by the distribution of their SOPs, which is the essential difference between two interference patterns. Therefore, it is necessary to insert polarizers with different orientations to examine SOPs of different vector beams. Here, we employ four polarizers with horizontal, vertical, and 45° and 135° orientations. When a polarizer is inserted, the intensity patterns of the vector beam show a fan-shaped extinction phenomenon, owing to the cylindrical symmetry in polarization and the same SOPs in the same azimuthal angle. For a specific vector beam, the fan-shaped extinction zones after inserting a horizontal polarizer are complementary to those after inserting a vertical polarizer (or inserting a 45° polarizer and a 135° polarizer). For RP and AP beams with the same topological charge and initial phase difference of *π*/2 when the same polarizer is inserted, there are also fan-shaped extinction phenomena and complementary fan-shaped extinction zones. The fan-shaped intensity patterns originate from the polarization interference between two beams carrying opposite topological charges, and the number of the fans are twice the absolute value of the topological charge |*l*|. Then, we also exhibit the generation of the vector beams with the topological charges of 2, 3, 4 and 5, respectively. The initial phases are set to 0 for simplicity. Obviously, despite the different topological charges, the fan-shaped extinction zones still stay complementary for the same vector beam after inserting two orthogonal polarizers, and the number of fans maintains twice the absolute value of the topological charge. In addition, the intensity patterns of the generated vector beams all occur in a dark spot located at the center because of the polarization singularity at the beam center. As the topological charge increases, the diameter of the central dark spot gradually expands.

**Figure 5:**
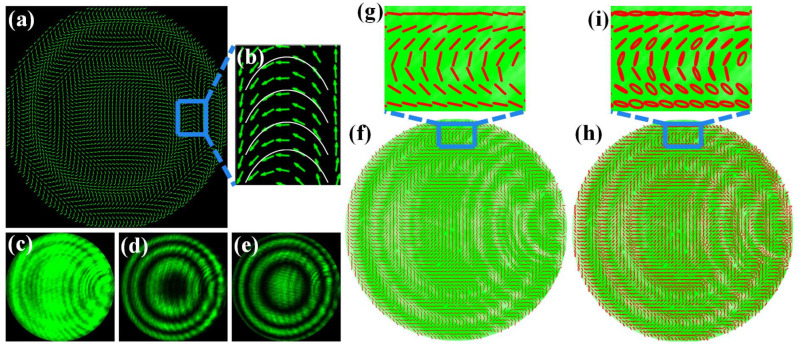
Generated vector beam with the desired phase profile *δ*_1_ of Equation (10). (**a**,**b**) The overall and local theoretical SOPs of the vector beam, respectively; (**c**–**e**) The intensity patterns of the vector beam after inserting no polarizer, horizontal polarizer and vertical polarizer, respectively; (**f**,**g**) The overall and local experimental SOPs with the linear components of the Stokes parameters of the vector beam, respectively; (**h**,**i**) The overall and local experimental SOPs with the full linear components of the Stokes parameters of the vector beam, respectively.

Next, we investigate the generation of the vector beams in the desired phase profile *δ*_1_ of Equation (10), as shown in Figure 5. As discussed above, the discrimination between the vector beam and the scalar beam cannot be distinguished by the intensity patterns without the analyzers. When two orthogonal polarizers are inserted, extinction zones of the vector beam are complementary too, as shown in Figure 5d,e. There are some minor defects in the quality of the beam, probably due to imperfect alignment of the experimental setup and uneven loss of the elements. Unlike the fan-shaped extinction zones described above, the extinction zones become ring-shaped as a result of the ring distribution of the SOPs. To feature specifically the distribution of the SOPs, we describe the vector beams with the aid of the Stokes parameters method, which calculates the linear polarization components and the full components for theoretical and experimental comparison and verification. Figure 5a describes theoretically the overall SOPs distribution of the vector beam, and Figure 5f,h describes experimentally the overall SOPs distribution on the intensity patterns with the linear components and the full components of the Stokes parameters, which are locally scaled up into Figure 5b,g,i, respectively. In particular, Figure 5a,b is not only the overall theoretical SOPs of the vector beam, but also the nanostructures arrangement of the wide-angle catenary metalenses. From the overall and local SOPs distribution, the SOPs distribution of the generated vector beam certainly matches the catenary nanopillars arrangement. The SOPs distribution with the full components of the Stokes parameters still stays catenary-shaped, although polarization states of some regions are not perfect linear polarization probably due to interference of the background light, spatial inhomogeneous beam intensity, or imperfectly aligned CCD figures. That is, we demonstrate that the generation of a space-variant vector beam with catenary-shaped polarization states is available.

**Figure 6:**
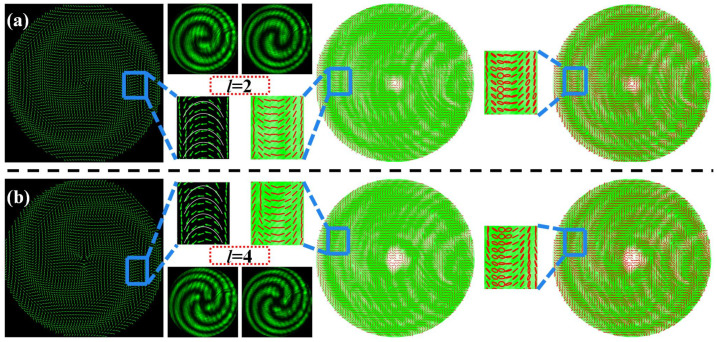
Generated vector beams with the desired phase profile *δ*_2_ of Equation (12). (**a**,**b**) Generated vector beams with topological charges of *l* = 2, and 4, respectively.

Finally, to validate the universal applicability of our method, we also investigate the generation of the vector beams with the topological charges of *l* = 2 and 4 in the desired phase profile *δ*_2_ of Equation (12), as shown in Figure 6. Different from all the cases described above, the extinction zones become spiral belt-shaped owing to the helical phase modulation introduced. Although the spiral belt-shaped extinction zones are still complementary for the same vector beam after inserting two orthogonal polarizers, the number of spiral belts is exactly equal to the absolute value of the topological charge, because the general quadratic phase superimposed with the helical phase introduces a factor of 1/2 in the geometric phase transformation. From six figures of the local SOPs distribution in the Figure 6, the SOPs distribution of the generated vector beam still conforms to the shape of the catenary.

## 5. Conclusions

In summary, we have theoretically proposed and experimentally demonstrated the generation of a space-variant vector beam with catenary-shaped polarization states with the aid of an experimental setup composed of an SLM and a 4-*f* system. The method may activate creative ideas for researching new effects and phenomena of complete vector beams, owing to its great flexibility and good universal applicability. In addition, these vector beams generated by combining metalens-type phase profiles, working in conjunction with femtosecond lasers or liquid crystals, are expected to be a huge boost in the micro/nano fabrication process for optical elements, such as lenses, gratings, and liquid crystals, and have many potential and intriguing applications in the micro/nano material processing, liquid crystal elements fabrication and optical micro-manipulation, and so on.

## Data Availability

Not applicable.
